# Case Report: Successful treatment of a case of Lynch syndrome with double primary ovarian and rectal cancer

**DOI:** 10.3389/fonc.2025.1534979

**Published:** 2025-10-07

**Authors:** Zhengliang Yu, Gang Yang, Jing Yue, Haiyan Liang, Man Yi, Yong Luo, Haixiao Fu, Zhenran Wang, Zhiyuan Jian, Yi Gao

**Affiliations:** ^1^ Department of Gastroenterology Surgery, The First Affliated Hospital of Guilin Medical University, Guilin, Guangxi, China; ^2^ College of Medical Laboratory Science, Guilin Medical University, Guilin, Guangxi, China; ^3^ Department of pathology, The First Affliated Hospital of Guilin Medical University, Guilin, Guangxi, China

**Keywords:** Lynch syndrome, high MSI and TMB, mispaired repair proteins, immunotherapy, ovarian and rectal cancer

## Abstract

**Background:**

Lynch syndrome (LS), previously known as hereditary nonpolyposis colorectal cancer (CRC), is an autosomal dominant disorder characterized by germline variants in the mismatch repair (MMR) gene (e.g., MLH1 and MSH2) with microsatellite instability (MSI), which leads to the development of CRC in 80% of cases with LS. Proximal colon is always involved in LS. LS is accompanied by an increased risk of developing glioblastoma, gastric cancer, and colorectal, endometrial, urothelial (ureteral and bladder), small intestinal, ovarian, biliary tract, and skin tumors (keratoacanthomas and sebaceous adenomas). The U.S. Food and Drug Administration has approved the use of pembrolizumab in the treatment of solid tumors with MMR defects or high MSI. Studies have shown that CRCs with MMR pathway loss-of-function variants respond favorably to PD-1 blockade immunotherapy.

**Case presentation:**

In this study, we report a case of LS in a 39-year-old female patient with concurrent ovarian and rectal adenocarcinoma. She showed high MSI, “pathogenic” germline variants in the MSH2 gene, and high tumor mutation burden. As a treatment modality, we chose a combination of immune checkpoint inhibitors, chemotherapy, and surgery and achieved a clinical complete response.

**Conclusion:**

This report is aimed at providing a reference for the diagnosis and treatment of tumors related to lynch syndrome, highlighting the diagnostic process of LS, and reporting treatment strategy of tumors related to lynch syndrome with the combination of immune checkpoint inhibitors, chemotherapy, and surgery.

## 1 Introduction

The pathogenesis of Lynch syndrome (LS) mainly results from variants in the genes of a group of mismatch repair (MMR) which encodes a group of proteins including MLH1, MSH2, MSH6, and PMS2. In addition, variants in the EPCAM gene, an upstream gene of MSH2, also cause defective MSH2 expression (1). MMR can repair single-base mismatches formed after DNA replication as well as insertion or deletion variants occurred during the replication of small repetitive sequences. When variants occur in the MMR gene, the cell loses its ability to recognize and repair gene mismatches, and these errors preferentially accumulate in microsatellite regions of the genome, causing genes to mute and subsequently resulting in microsatellite instability (MSI) (2). The accumulation of variants then affects the activity of some important functional proteins in the cell, thus causing tumorigenesis (3). Investigations in LS family lines have revealed that about 85% of the examined variants are MLH1 and MSH2 variants, and the remaining are MSH6 and PMS2 variants (4). Compared to sporadic colorectal cancer (CRC), the onset of Lynch syndrome occurs early in younger age (mean age of onset, 45 years) and carries a significantly higher incidence of concurrent or heterochronic multiple primary cancers (5).

Among the options for treating patients with LS, immune checkpoint inhibition is one of the most widely discussed immunotherapies. Patients with dMMR solid tumors are reportedly sensitive to immunosuppressive agents, and for programmed death protein-1 and its ligands (PD-1 and PD-L1) (6). Recently, high tumor mutation burden (TMB) has also been widely discussed by scholars as an indicator of the efficacy of immunosuppressive therapy. In the KEYNOTE-158 study, patients with TMB-H were found to have higher objective remission rates with pembrolizumab than those with TMB for 10 tumors (7). Herein, we present a case of LS in a patient with both ovarian and rectal adenocarcinoma who achieved R0 resection and long-term survival following MDT-guided comprehensive treatment (**Supplementary Figure S1**).

## 2 Case report

**Figure 1:**
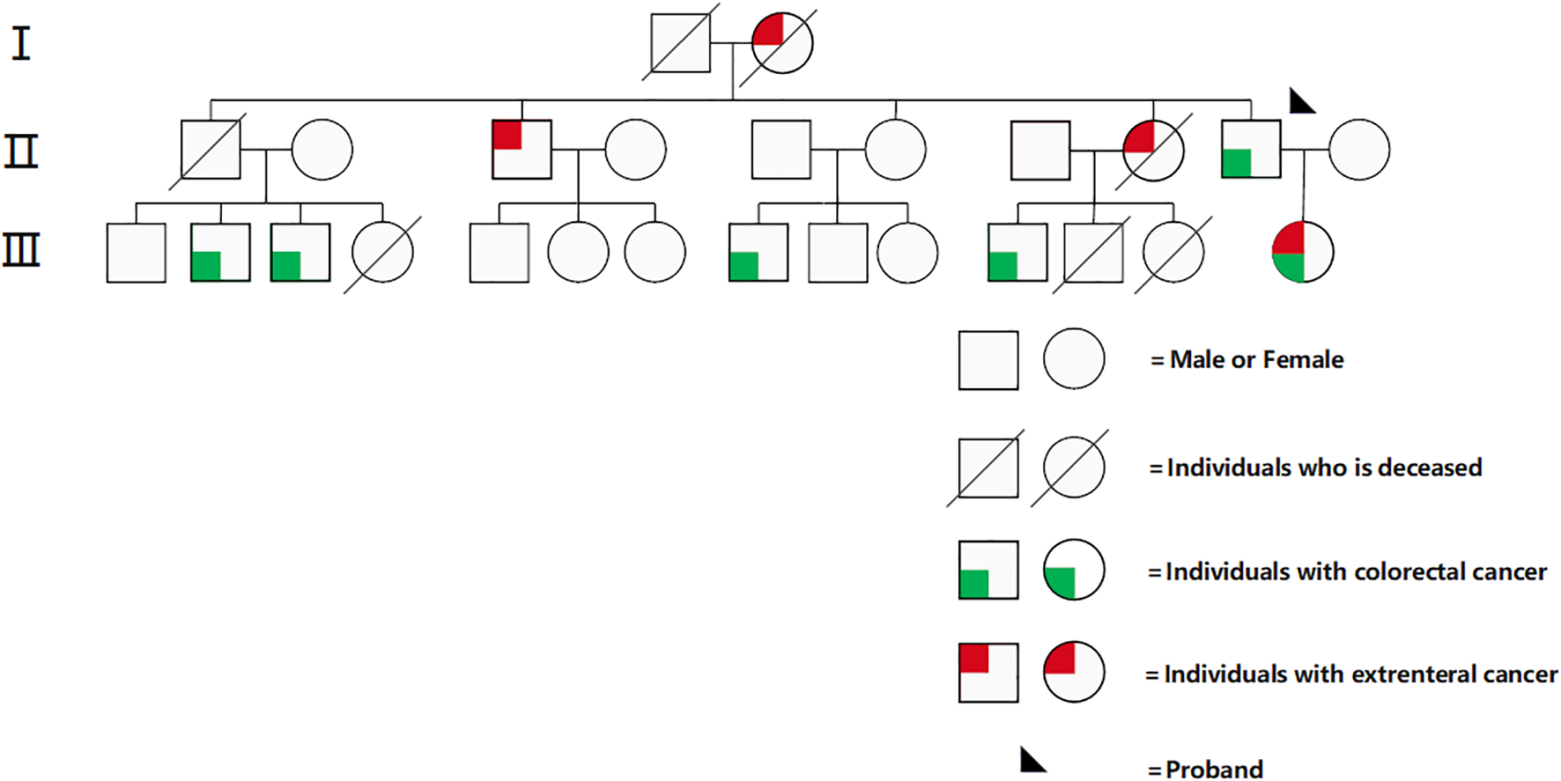
Family tree and incidence of LS.

A 39-year-old female patient who presented to the Affiliated Hospital of Guilin Medical University with “abdominal pain for half a month” was admitted on January 28, 2022. She had a previous successful pregnancy wherein she underwent a cesarean section; she typically had a regular menstrual cycle of 26 days, with bleeding lasting 4–6 days (last menstrual period: 31st December, 2021). She had a positive family history for cancer. Her second, third, fifth, and seventh cousins had intestinal cancer; her second uncle had hepatocellular carcinoma; her second aunt had cervical and gastric cancers; and her father had intestinal cancer (**Figure 1**). Gynecological examination revealed a palpable right adnexal mass with poorly defined borders, pressure pain, and poor mobility. On 28th January, 2022, color ultrasound (transvaginal uterine and adnexal ultrasound) showed a solid mass measuring ~97 mm × 66mm × 67mm on the right side of the pelvis and a cyst in the left adnexa. Pelvic magnetic resonance enhancement showed a large pelvic mass, and malignant tumor of epithelial origin of the right adnexa was considered likely. A space-occupying lesion was noted in the upper rectum. Gastroscopy showed chronic non-atrophic gastritis. On 29th January, 2022, the remarkable laboratory findings were as follows: β-HCG < 0.200 mIU/mL, CA125: 1090.00 U/mL↑, human epithelial protein 4: 146.900 pmol/L↑; white blood cell count: 14.220 × 109/L↑, and hemoglobin concentration: 107.000 g/L↓. She underwent colonoscopy on 4th February, 2022, which revealed the upper rectal wall mass, and the pathology results of the subsequent biopsy suggested highly differentiated tubular adenocarcinoma. Her immunohistochemistry results were as follows: CK7 (-), CK20 (+), VILLIN (+), CDX-2 (+), PAX-8 (-), and DES individual mesenchymal cells (+). Factoring in the immunohistochemistry results, the origin was established to be gastrointestinal. Immunohistochemical analysis of MMR proteins in rectal pathology revealed MSH-2 (-), MSH-6 weak (+), PMS-2 (+), and MLH-1 (+), suggestive of dMMR. To clarify the origin of the pelvic mass, ultrasound-guided puncture biopsy of the pelvic mass was performed on 15th February, 2022, and the pathology suggested low-differentiated carcinoma with extensive necrosis, and combined with immunohistochemistry findings, high-grade adenocarcinoma originating from the female reproductive system was considered. Immunohistochemistry results showed cancer cells CK7 (+), PAX-8 (+), WT-1 (individual weak +), CK20 (-), CDX-2 (-), SATB2 (-), P53 (strong +, 60-70%), Ki67 (+, 50%), ER (-), PR (-), and P16 (+). The patient presented with dual primary adenocarcinomas of the rectum and ovaries, and considering her family history and current medical history, we strongly suspected LS. On 23rd February, 2022, high-throughput sequencing genetic testing was performed, which revealed microsatellite analysis results of MSI-H with a tTMB value of 97.8 variants/Mb on somatic tissue and a “pathogenic” germline mutation in the MSH2(c.942 + 3A>T) gene on blood. No variants affecting protein function were detected in other genes(including *MLH1, MSH2, MSH6, PMS2*, *EPCAM* and *POLE/POLD1*). PD-L1 combined positive score (CPS) was not tested in this patient.

The patient’s primary diagnosis at this time was double primary ovarian cancer and rectal adenocarcinoma with LS. Enhanced CT of the abdomen showed a rounded hypodense shadow in liver S5, with blurred borders and a diameter of ~10 mm. Multiple enlarged and inhomogeneously reinforced lymph nodes adjacent to the abdominal aorta were visualized, with the largest one measuring ~24 mm × 21mm. A mass-like hypodense shadow measuring ~83 mm × 75mm × 117mm with a clear lobulated border was seen in the pelvis. The upper rectal wall was limitedly thickened, with a more pronounced area of about 15mm. Due to the complexity of the patient’s condition, we conducted a multidisciplinary team (MDT) consultation on 4th March, 2022, the outcome of which was a clear diagnosis of LS (adenocarcinoma of the rectum and ovary). Considering the large size of the tumor, the possibility of metastasis to the liver and para-aortic lymph nodes, and the possibility of hepatic metastasis, it was not possible to perform radical surgical resection. Based on the 2022 edition of the NCCN guidelines and clinical research data at home and abroad, it is recommended that patients with unresectable or metastatic advanced MSI-H/dMMR solid tumors can be treated with immune checkpoint inhibitors. Therefore, we used translational therapy with the following regimen: carboplatin 400 mg (ivgtt), paclitaxel 180 mg (ivgtt), and sindilizumab 200 mg (ivgtt); this was repeated every 3 weeks for four cycles of chemotherapy.

**Figure 2:**
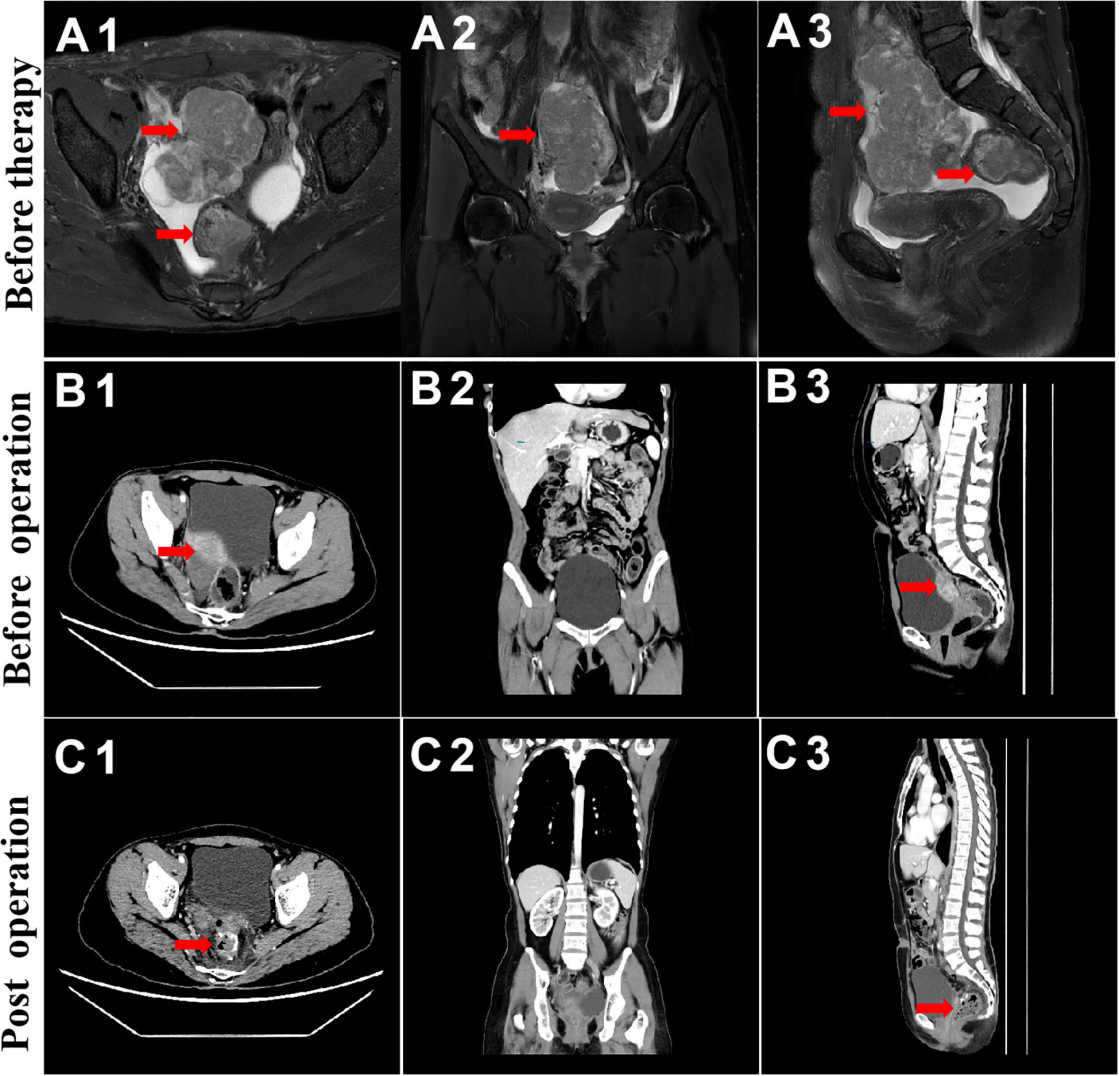
MRI and CT evaluation of ovarian and rectal tumors. From top to bottom, before chemotherapy and immunotherapy **(A)**, before surgical treatment **(B)**, and during 3-week postoperative follow-up **(C)**.

On 2nd June, 2022, chest and abdominal enhancement CT was repeated, which suggested the presence of a mass on the basis of an abnormal single shadow on the right margin of the uterus. The mass measured ~37 mm × 26mm, which was significantly reduced compared with the findings of the previous CT scan. Additionally, there was a significantly reduced thickening of the upper rectal intestinal wall (**Figure 2**), which was previously thicker by about 8mm and involved an area of about 25mm. A rounded low-density nodule measuring ~14 mm × 11mm was seen in liver S5. Lymph node shadow was seen next to the abdominal aorta, which had a diameter of about 8mm and was significantly reduced compared with the previous CT findings.

**Figure 3:**
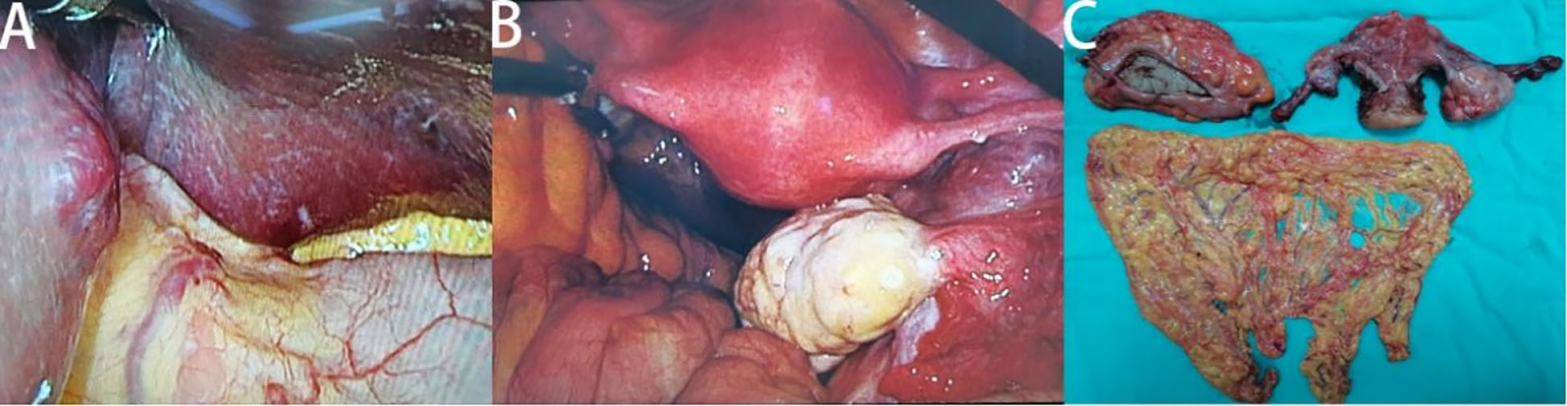
Intraoperative and postoperative specimen images of patients. Hemangiomas in the left lobe of the liver **(A)**, cancer in the right ovary **(B)** and postoperative specimen display **(C)**.

**Figure 4:**
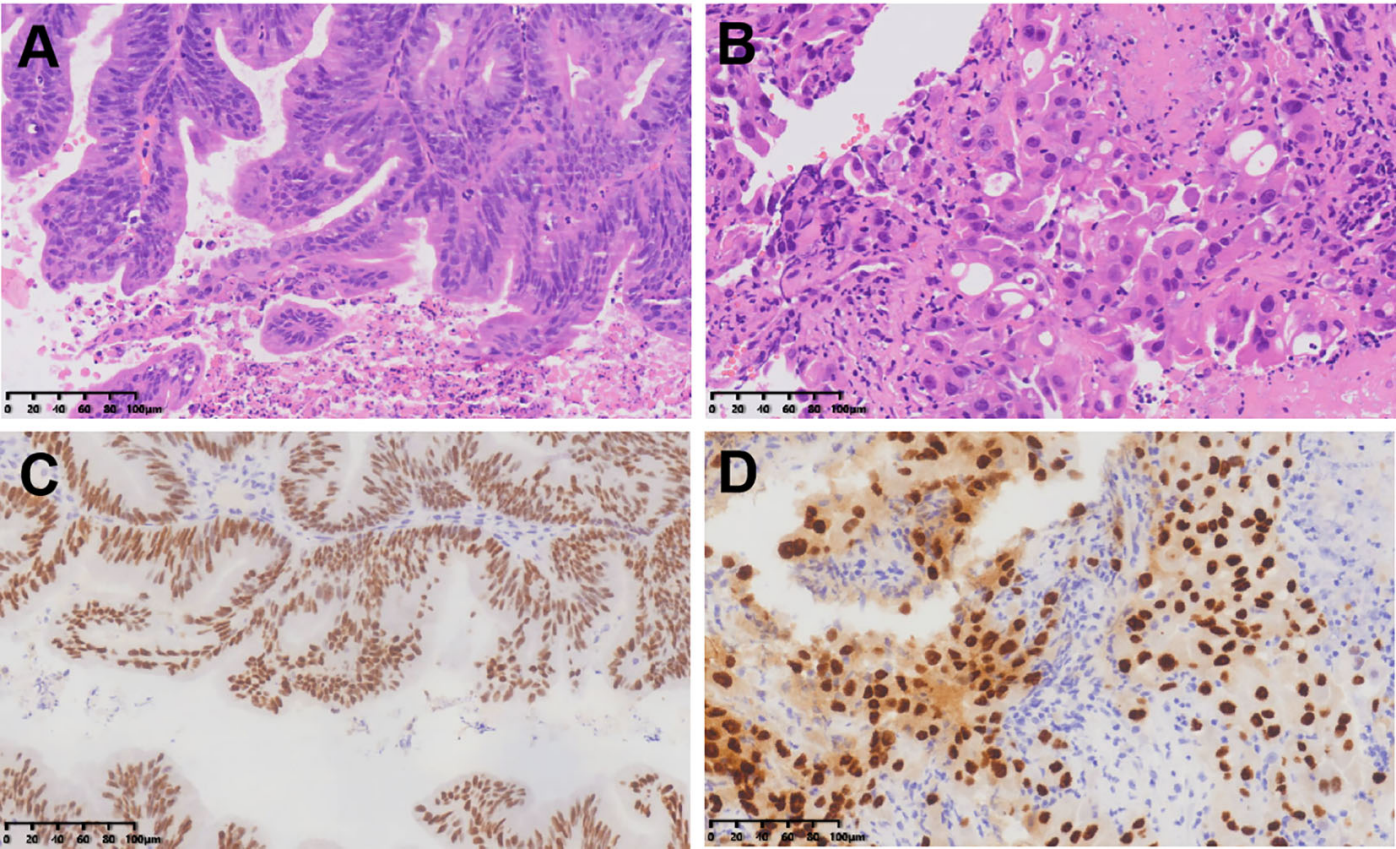
HE staining of rectal adenocarcinoma **(A)** and ovarian adenocarcinoma **(B)**; Immunohistochemical staining of intestinal CDX-2 **(C)** and ovarian PAX8 **(D)**.

To ensure good outcomes of the transformation and to re-evaluate the possibility of radical resection of both primary foci, we had another MDT consultation on 9th June, 2022, with the following treatment opinion: laparoscopic exploration at first, with the possibility of gastrointestinal, hepatobiliary, and gynecologic surgery, if necessary. The patient underwent laparoscopy under general anesthesia on 14th June, 2022, and the intraoperative lesions in the left lobe of the liver were identified as hemangiomas (**Figure 3A**). A 3 × 4cm mass was seen in the right ovary (**Figure 3B**), and a slightly toughened local intestinal wall was seen in the upper part of the rectum, and this part measured ~2 cm in length. No metastatic nodules were seen on the surface of the diaphragm, intestinal tubes, peritoneum, and omentum. Laparoscopic radical rectal cancer surgery, bilateral adnexal resection, and total omentectomy were performed (**Figure 3C**). Postoperative pathology revealed the following: (rectal cancer) intramucosal cancer (moderately to highly differentiated adenocarcinoma); residual tumor size: ~0.3 cm × 0.3cm × 0.1cm, with no clear intravascular embolus or neural invasion; no cancer in upper and lower margins; no metastasis of the eight mesenteric lymph nodes (0/8); visible gravel body at the perineural surface of one of the lymph nodes. Immunohistochemistry results with intestinal CDX-2 staining were as follows (**Figure 4C**): cancer cells CDX-2 (+), Pax-8 (-), uterine and bilateral adnexa, high-grade adenocarcinoma (originating from the female reproductive system) with extensive necrosis in the right ovary, and 0.1-cm thick cancerous residue. Gravel body formation was seen in the surrounding tissues, with no clear intravascular embolus or nerve invasion, and no tumors were seen in the soft tissues of the parietal uterus bilaterally. Finally, no specific lesions were seen in the left ovary and bilateral fallopian tubes. Immunohistochemistry results were as follows (**Figure 4D**): cancer cells Pax-8 (+), P16 (+), CK7 (+), and CDX-2 (-). Postoperative follow-up CT performed on 6th July, 2022 suggested no significant changes in bilateral lung micronodules (LMs). There were no significant changes in the liver S5 segmental lesion; the rectum and abdominopelvic cavity showed postoperative changes with no obvious abnormalities.

Postoperatively, the previous regimen of carboplatin 400 mg (ivgtt), paclitaxel 180 mg (ivgtt), and sindilizumab 200 mg (ivgtt) every 3 weeks was continued for a total of four cycles, according to the MDT consultation. On 8th September, 2022, the patient was started on sindilizumab immunomaintenance therapy [sindilizumab 200 mg (ivgtt)], which was administered every 3 weeks for a total of 4 cycles. In December 2022, she complained of generalized itching of the skin, and liver function test findings revealed elevated bilirubin and aminotransferase levels. We speculated that the drug was causing liver damage. The patient refused to continue immunotherapy. Thus, we discontinued the immunotherapy and started her on a combination of diammonium glycyrrhizinate and isoglycyrrhizinate magnesium to protect the liver. Then, bilirubin and aminotransferase normalized. On 27th April, 2023, a review of her PET-CT showed (i) no obvious recurrence signs in the operation area, (ii) small lymph nodes next to the abdominal aorta showing no increase in FAPI expression, (iii) hepatic S5 hemangiomas, and (iv) multiple tiny nodules in both lungs, not accompanied by an increase in FAPI expression. On 14th November, 2023, a review of her enhanced thoracic and abdominal CT showed no obvious abnormalities of the gastrointestinal tract, which was consistent with postoperative changes typically noted in patients with rectal cancer. There were no obvious abnormalities in liver and kidney function or in CEA, CA125 (**Supplementary Figure S2**), and CA199 levels. The patient returned to the hospital for regular follow-up more than 2 years after the initial diagnosis, at which point no signs of tumor recurrence or metastasis were noted.

## 3 Discussion

The current international clinical standards and protocols for screening for LS mainly include the Amsterdam II criteria and the revised Bethesda criteria. According to the Bethesda criteria, the following patients should be screened for LS: 1. patients with CRC who are <50 years of age at first presentation; 2. patients (regardless of age) with concurrent or heterochronous CRC or other LS-related tumors; 3. patients <60 years of age with CRC and histology suggestive of a high degree of MSI (MSI-H); 4. patients with at least 1 first-degree relative diagnosed with CRC or LS-related cancer before the age of 50 years; and 5. patients with 2 first- or second-degree relatives with CRC or LS-related cancer (8).The sensitivity of screening LS patients based on the above clinical criteria is low, with the advent of testing modalities such as immunohistochemistry and microsatellite instability testing, genetic testing, etc., for LS we have a higher detection rate than ever before.

The age of LS-associated tumors in our patient was 39 years, and her second, third, fifth, and seventh cousins had bowel cancer. Her second uncle had liver cancer, and her second aunt had cervical and gastric cancer. Her father had bowel cancer, which, combined with her family genetic history, made us strongly suspect LS. Then, MMR immunohistochemistry results for rectal adenocarcinoma revealed that the tumors had dMMR. MMR genetic examination is the gold standard for LS, and the final results suggested features of MSI-H, TMB-H on somatic tissue, and a “pathogenic” germline mutation in the MSH2 gene on blood. The results of the KEYNOTE-158 and KEYNOTE-177 tests showed that due to the clinical benefit of PD1 inhibitors in patients with unresectable or metastatic MSI-H/dMMR non-CRC, the FDA approved pembrolizumab monotherapy in June 2020 for advanced TMB-H (≥ 10 mut/Mb) that has progressed after standard therapy. Although the NCCN guidelines recommend pembrolizumab monotherapy as first-line therapy for patients with unresectable or metastatic MSI-H/dMMR non-CRC, Sindilizumab had been adopted in China based on its demonstrated efficacy in ORIENT-16 trial (9). Our choice of Sindilizumab was further supported by the local poor medical conditions and its superior cost-effectiveness in the Chinese healthcare system in 2022. Moreover, given the large tumor burden of ovarian adenocarcinoma and its sensitivity to chemotherapy, the MDT-Guided Comprehensive Treatment chose the treatment regimen of immunotherapy combined with chemotherapy. Therefore, we chose to give the patient preoperative systemic chemotherapy and immunotherapy; consequently, we achieved effective remission of both the primary tumor and metastatic lymph nodes, which made the ensuing surgical R0 resection possible. Postoperatively, the same regimen of chemotherapy plus immunotherapy was continued, and no signs of tumor recurrence or metastasis were noted at the 39-month postoperative follow-up. In conclusion, we herein reported a case of a 39-year-old female patient having primary ovarian and rectal adenocarcinoma with LS who demonstrated complete response to chemotherapy plus sindilizumab. The high MSI and TMB in this patient may have contributed to the favorable response to immunotherapy.

## 4 Conclusion

This report details the diagnostic and therapeutic course of a patient with LS having double primary ovarian and rectal adenocarcinoma with an aim to provide a reference for the diagnostic and therapeutic strategy of double primary and even multiple primary LS tumors, particularly MMR-deficient/MSI-H/highly mutated tumors.

## Data Availability

The datasets presented in this article are not readily available because of ethical and privacy restrictions. Requests to access the datasets should be directed to the corresponding authors.

## Supplementary material

Supplementary Figure 1 Flow chart of the patient’s diagnosis and treatment.

Supplementary Figure 2 Trend graph of patients’ tumor marker CA125 change.
