# Supplementary material for: Case Report: Successful treatment of a case of Lynch syndrome with double primary ovarian and rectal cancer
Source: Front Oncol. 2025 Oct 7;15:1534979. doi: 10.3389/fonc.2025.1534979 (PMC12537393; doi:10.3389/fonc.2025.1534979)
Supplement: Supplementary Figure 1 — Flow chart of the patient’s diagnosis and treatment. [file DataSheet1.docx]

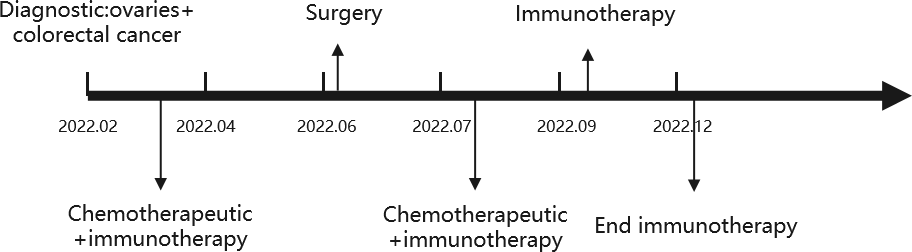


**Supplementary Figure S1. Flowchart of the patients’ diagnosis and treatment**

**
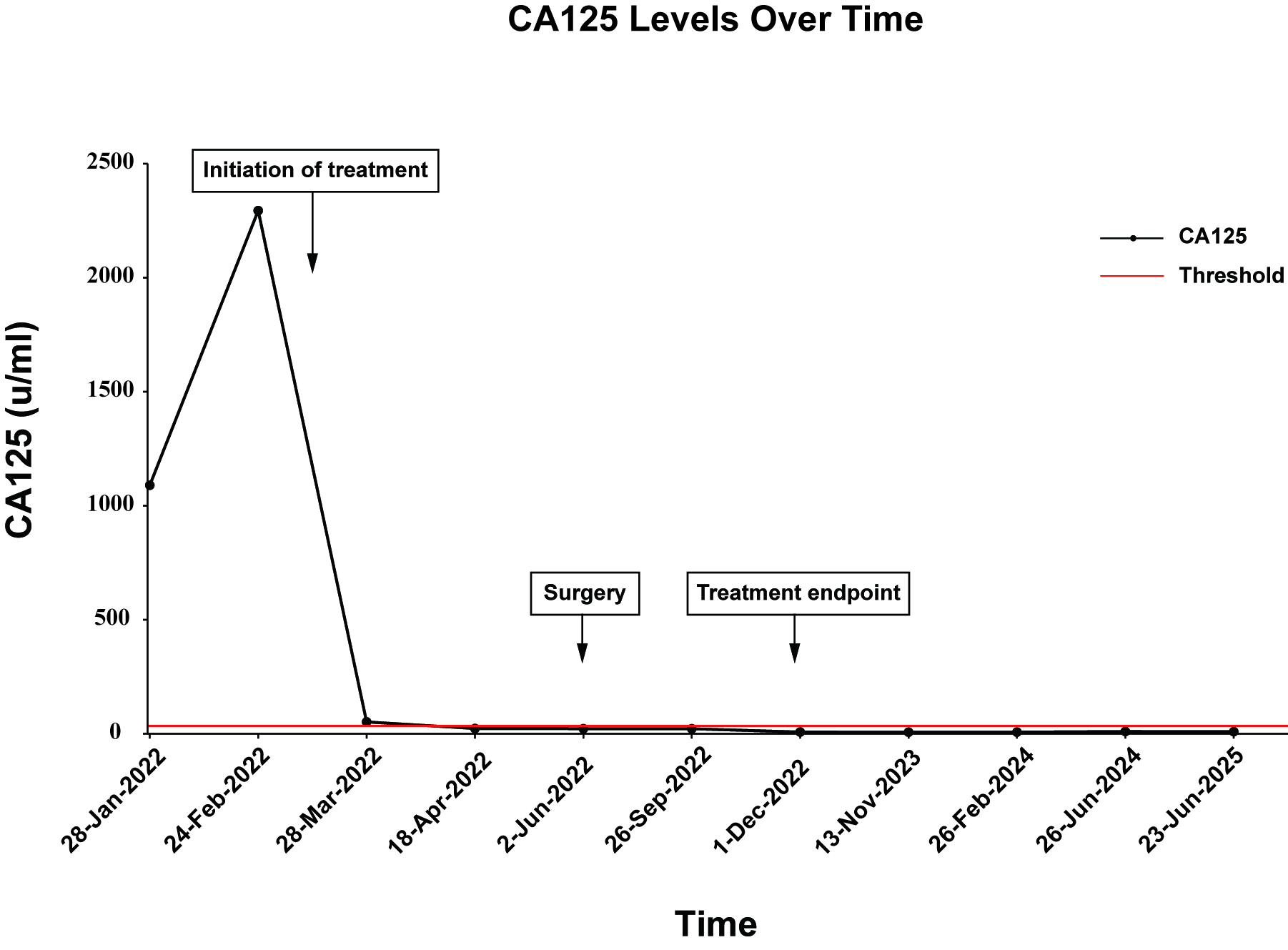
Supplementary Figure S2. Trend graph of changes in patients’tumor marker CA125**
